# Organic Farming Enhances Diversity and Recruits Beneficial Soil Fungal Groups in Traditional Banana Plantations

**DOI:** 10.3390/microorganisms12112372

**Published:** 2024-11-20

**Authors:** Maria Cristina O. Oliveira, Artur Alves, Carla Ragonezi, José G. R. de Freitas, Miguel A. A. Pinheiro De Carvalho

**Affiliations:** 1 ISOPlexis, Center for Sustainable Agriculture and Food Technology, University of Madeira, 9020-105 Funchal, Portugal; maria.oliveira@staff.uma.pt (M.C.O.O.); jgrfreitas@staff.uma.pt (J.G.R.d.F.); miguel.carvalho@staff.uma.pt (M.A.A.P.D.C.); 2 ARDITI—Agência Regional para o Desenvolvimento da Investigação, Tecnologia e Inovação, Caminho da Penteada, 9020-105 Funchal, Portugal; 3 CESAM—Centre for Environmental and Marine Studies, Department of Biology, University of Aveiro, Campus Universitário de Santiago, 3810-193 Aveiro, Portugal; artur.alves@ua.pt; 4 Centre for the Research and Technology of Agro-Environmental and Biological Sciences (CITAB), Inov4Agro—Institute for Innovation, Capacity Building and Sustainability of Agri-Food Production, University of Trás-os-Montes and Alto Douro, 5000-801 Vila Real, Portugal; 5 Faculty of Life Sciences, University of Madeira, Campus da Penteada, 9020-105 Funchal, Portugal

**Keywords:** mycobiome, banana soil fungi, functional groups, organic farming, fungal diversity

## Abstract

This study investigates the impact of organic (OF) and conventional farming (CF) on soil fungal communities in banana monoculture plantations on Madeira Island. We hypothesized that OF promotes beneficial fungal groups over harmful ones, sustaining soil health. Soil samples were collected from six plantations (three OF and three CF) for ITS amplicon sequencing to assess fungal diversity. Results showed that OF significantly enhanced fungal alpha-diversity (Shannon–Wiener index) and Evenness. The phylum Ascomycota dominated OF systems, while Basidiomycota prevailed in CF. *Mortierella*, a beneficial genus, was abundant in OF and is observed in CF but was less evident, being the genus *Trechispora* the most well represented in CF agrosystems. Additionally, OF was associated with higher soil pH and Mg levels, which correlated positively with beneficial fungal groups. Functional analysis revealed that OF promoted saprotrophic fungi, crucial for the decomposition of organic matter and nutrient cycling. However, both systems exhibited low levels of arbuscular mycorrhizal fungi, likely due to high phosphorus levels. These findings suggest that organic practices can enhance soil fungal diversity and health, although attention to nutrient management is critical to further improving soil–plant–fungi interactions.

## 1. Introduction

Bananas are one of the most widely produced, traded, and consumed fruits worldwide. This fruit crop plays a vital role in world agricultural production, especially for smallholders [1]. On Madeira Island, the banana crop was introduced in the 16th century and gradually became a major source of wealth [2]. However, only in the 20th century was the crop traded abroad [3]. Madeira produces bananas with unique characteristics, known for their intense flavor and aroma [2,3]. This is due to environmental conditions such as proximity to the sea, sun exposure, mild climate throughout the year, and soil fertility combined with the selection of clones with the best quality and yields [3,4]. They are predominantly cultivated on Madeira’s south coast, up to 300 m above sea level [2].

According to regional statistics, bananas are among the most economically relevant fruit crops for Madeira. The latest agricultural census data account for 5.171 banana farms, representing 35% of the total agricultural area used for permanent crops [5]. In 2023, more than 85% of the production was shipped, mainly to mainland Portugal [6].

Because of the importance of this crop, traditional banana cropping systems in Madeira Island have been under monoculture for many years, most of them for more than 50 years. For most of the Banana plantations from Madeira, three varieties are used, namely ‘Robusta’, ‘Pequena anã’, and ‘Grande anã’. The growth of banana plants as monocultures increases the problems with diseases and pests [7]. For example, *Fusarium* wilt became a major concern for producers in several regions, including Madeira Island [7,8,9]. The fungus can invade the plant, penetrating the cell wall of the roots or through wounds or injury sites of the roots, then it spreads all over the plant and sporulates, plugging the xylem vessels, creating a water deficit condition, and causing wilting of the host plant [9]. The only current effective strategy to control the disease is the planting of resistant cultivars, even though, owing to its high mutation rates and rapid co-evolution with the host, *Fusarium* wilt has likely bypassed host defenses and is now infecting resistant varieties [9]. In addition, the long-term cultivation of the same crop in the same soil may result in soil degeneration, with adverse effects on multiple abiotic and biotic indicators of soil health, including microorganisms with important functions in soil [10].

The importance of soil microorganisms has been recognized for a long time. Microorganisms are a driving force behind the soil processes that are crucial for sustaining agricultural production [11]. Fungi, in particular, can act as harmful pathogens [12,13], but also are the main decomposers of organic materials in soils [14,15], are important symbionts of plants [16], may contribute to maintaining soil structure and nutrient cycling [17] and interact with other organisms as biocontrol agents, like *Trichoderma* species [18]. However, only recently, the studies on soil microbial communities in agriculture have increased considerably due to the development of high-throughput sequencing technologies [19,20]. Previous research verified that agricultural intensification may change the diversity, composition, and functioning of microbial communities in soil, which can have a negative impact on the proper functioning of an agroecosystem due to their key role in soils [21,22]. Management practices used in organic farming, when compared to conventional practices, are considered to play beneficial roles in improving and preserving microorganisms’ diversity and biological activity of the soil [10,23], which contributes to sustaining elevated nutrient levels in the soil, boosting crop productivity and enhancing soil health [24]. In addition, some amendments may contribute to a decrease in the incidence or severity of diseases caused by soil-borne plant pathogens [25,26,27]. Notably, soil from organic practices shows an enrichment of specifical fungal genera known for their biocontrol properties, which enhances its disease-suppressive capabilities, making organic soil more effective in suppressing disease than soil from conventional systems [28]. Organic farming practices also prevent the loss of soil fertility through the maintenance of the structural stability of the soil, the cycling of nutrients, and the improvement of other physical and chemical properties of the soil [10,23]. In agrosystems under monoculture, organic farming can have a positive impact on soil health, although the cost of monoculture possibly increases over time [10]. On the other hand, conventional farming is based on the use of synthetic fertilizers and pesticides and on management practices that force productivity, but with cost for agroecological interactions, which exacerbate the imbalance of the agrosystems [23].

The differences in management practices and locations of banana plantations are shown to influence the soil’s edaphic conditions, thereby impacting its fungal diversity [29,30]. Little is known about the impact of organic amendments on soil fungal communities from monoculture banana plantations in Madeira Island. Hence, in this study, we hypothesize that organic farming practices used in these banana agrosystems are able to select important fungal functional groups over harmful ones, contributing to sustaining the soil health and fertility of an agroecosystem, in contrast to conventional farming practices. The present study aimed to analyze and compare the soil fungal communities, with a focus on functional groups, of traditional banana plantations under organic and conventional farming.

## 2. Materials and Methods

### 2.1. Site Description and Soil Sampling

Soil samples were collected between May and June of 2020 from 6 banana plantations (agrosystems) in monoculture along the south coast of Madeira Island, differing in the management system (MS) adopted; 3 of them are under organic farming (OF) and the other 3 are under conventional farming (CF) (Table 1). For CF and OF, traditional cultivation methods are used. The cultural practices of bananas, for both systems, include cutting the leaves (for plant cover), fragmenting the pseudo-stems to speed up their decomposition in the soil, and on many farms, they still use flood irrigation, generally being non-mechanized systems. Conversion from CF to OF takes about 3 years, according to national guidelines. Both management systems receive fertilization; however, CF is of synthetic origin, and OF is of organic origin. Both obtain P fertilizer via fertigation or directly in the soil. The banana plants did not indicate any symptoms related to diseases during the sampling. Sampling was performed according to Paetz and Wilke [31] with adaptations. Briefly, soil samples were collected in a zigzag pattern across the field from 15 points, approximately 10 cm deep, less than 50 cm away from banana plants. The 15 sub-samples were pooled and well mixed into a clean plastic bag to obtain a sample representative of the soil of each agrosystem (n = 6) and immediately transported to the laboratory. Each soil sample was then divided into two parts; one part was distributed into aliquots and stored at −20 °C for molecular analysis, and the other part was air-dried, grounded, and sieved (2 mm) for determination of macronutrients (NO_3_-N, NH_4_-N, P, and K) and micronutrients (Ca, Mg, Na, Cu, Zn, Mn, and Fe), pH, organic matter (OM), cation exchange capacity (CEC), and saturation degree (SD) at the Agricultural Quality Division of Laboratory and Agri-Food Research Services in Camacha, Madeira, Portugal. Soil physicochemical properties were determined previously according to Portuguese legislation; for details, and for pH, OM, CEC, and macronutrients, see Ragonezi et al. [32], and see Temminghoff and Houba [33] for SD and micronutrients (for detailed soil physicochemical properties determination, see Supplementary Material).

### 2.2. DNA Extraction and ITS Amplicon Sequencing

Total genomic DNA was extracted from soil samples, according to Yeates et al. [34], with adaptations. For each agrosystem, 6 tubes with 250 mg (dry weight) of soil and ±250 mg of glass beads (≤106 µm, acid-washed; Sigma-Aldrich, St. Louis, MO, USA) were filled with 400 µL of extraction buffer (100 mM Tris-HCl, pH 8.0; 100 mM sodium EDTA, pH 8.0; 1.5 M NaCl) and vortexed for 10 min. Then, 25 µL of sodium dodecyl sulfate (10%) was added to each tube and mixed for 5 s. Tubes were incubated (65° C for 1 h) and centrifuged (16,000× *g* for 10 min), and the supernatants were transferred to new tubes. The soil pellets were re-extracted with 400 µL of extraction buffer, incubated (65 °C for 10 min), and centrifuged (16,000× *g* for 10 min). Supernatants were added to the tubes of the first extraction, then a half-volume of polyethylene glycol (30%)/sodium chloride (1.6 M) was added to each tube and incubated for 2 h at room temperature. After centrifugation (16,000× *g* for 20 min), pellets were resuspended in 140 µL of Tris-EDTA (10 mM Tris-HCl, 1 mM sodium Ethylenediaminetetraacetic acid, pH 8.0). Potassium acetate (7.5 M) was added to a final concentration of 0.5 M. Tubes were incubated (4 °C for 5 min) and then centrifuged (16,000× *g* for 30 min). Phenol/chloroform/isoamyl alcohol (25:24:1) was added and mixed gently, then tubes were centrifuged (2500× *g* for 15 min), and the aqueous phase was transferred to new tubes. DNA was precipitated by adding 1.5 volume of absolute ethanol and incubated overnight at −20 °C. DNA was pelleted (16,000× *g* for 30 min) and resuspended in TE (25 µL). The DNA obtained by the extraction of 6 aliquots of each soil sample was pooled and gently mixed in the end (n = 6). The concentration and purity of DNA were measured with NanoDrop^®^ 2000c Spectrophotometer (ThermoFisher Scientific, Waltham, MA, USA). DNA extracts were purified using an ExtractME DNA clean-up kit (Blirt, Gdańsk, Poland) and preserved at −20 °C.

PCR amplification of the ITS1 region of the fungal nuclear rRNA gene cluster was achieved using primers ITS5-1737F (5’ GGAAGTAAAAGTCGTAACAAGG 3’) and ITS2-2043R (5’ GCTGCGTTCTTCATCGATGC 3’) [35]. Amplicon cleaning, library preparation, and following sequencing were performed at Novogene (Cambridge, UK) through Illumina Sequencing NovaSeq technology (Illumina, San Diego, CA, USA) (paired-end reads of 250 nt). DNA samples were analyzed in duplicate (n = 12, 50 K tags per sample).

### 2.3. Bioinformatics Pipeline

Paired-end reads were demultiplexed and then truncated by cutting the barcodes and primer sequences with Cutadapt (v3.3) [36]. The software FLASH (v1.2.11) was used to merge the reads (default parameters, except for fragment length that was set to 300 and a maximum mismatch density to 0.1) [37]. Quality control of raw tags was checked using fastp (v0.20.0) software (qualified quality phred: ≥Q19; unqualified percent limit: 15%) [38]. Detection and removal of chimeric sequences was performed using Vsearch (v2.15.0) software [39]. The reads were denoised with DADA2 (v4.2.0) [40] (default settings) in QIIME2 (v2020.6.0) software [41]. The size of the obtained Amplicon Sequence Variants (ASVs) ranged between 200 and 400 bp. ASVs with less than 5 reads were removed, and the final set of ASVs was compared with the UNITE database (v9.0) [42] for species annotation, using a sklearn-based taxonomy classifier [43]. The absolute abundance of ASVs was normalized using a standard sequence number corresponding to the sample with the least sequences (50,735 high-quality reads). The normalized data were used for the following analyses of alpha- and beta-diversity and functional communities.

### 2.4. Data Analysis

Alpha- and beta-diversity were analyzed using R software (v4.3.1). The drawing of the rarefaction curves and calculation of Richness, Shannon–Wiener diversity, and Evenness indices were performed using the package vegan (v2.6-6.1) [44], and boxplots were drawn with package ggplot2 (v3.5.1) [45]. Permutational multivariate analysis of variance (PERMANOVA) based on the Bray–Curtis distance for fungal functional communities was performed using the functions “vegdist” and “adonis2” from package vegan, and the function “betadisper” was used to test the homogeneity of groups dispersions, through Marti Anderson’s PERMDIST2 procedure. Constrained Analysis of Principal Coordinates (CAP) based on Bray–Curtis dissimilarity distance matrix of soil fungal functional communities was ordinated using the function “capscale” from package vegan and drawn using the package ggplot2. Bar charts for communities’ composition were drawn on Excel 2019 (Microsoft Corporation, Redmond, WA, USA v1808). ASVs were assigned to a probable or highly probable ecological function using the database FUNGuild [46], and boxplots were drawn on R using the package ggplot2.

The normality and homogeneity of variance assumptions of the variables under study were evaluated in the statistical package for the social sciences (SPSS, IBM SPSS Statistics, Armonk, NY, USA v27.0) for Windows using Shapiro–Wilk and Levene’s tests. For comparisons of means between management systems, Student’s *t*-test or Welch’s *t*-test was performed depending on if homogeneity of variances was assumed or not, and when normality assumptions were not verified, the Mann–Whitney U test was used. Correlation analysis was performed with Spearman’s coefficient, and the graph was drawn with package corrplot (v0.92).

## 3. Results

### 3.1. Effect of Management Systems on Soil Physicochemical Properties

Comparing the means of soil physicochemical properties for organic farming (OF) and conventional farming (CF) through Student’s *t*-test or Mann–Whitney U test (Table 2), we verified that pH and Mg were the properties that were mainly affected by the management practices adopted. OF agrosystems showed pH around 6.57 and Mg around 12.77 meq/100 g, while CF presented values of 4.56 and 6.00, respectively. The micronutrient Ca also showed higher values in OF soils; however, the difference was not statistically significant. For all the other properties, it was found that they had similar values in soils from both management systems (Table 2).

### 3.2. Soil Fungal Diversity in OF and CF Management Systems

**Figure 1:**
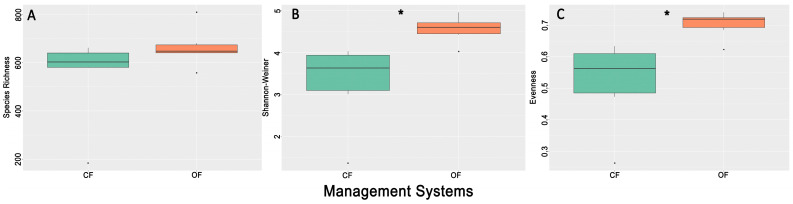
Variation of α-diversity indices in both management systems, conventional farming (CF) and organic farming (OF). The significant difference between CF and OF is labeled with (*), Mann–Whitney U test, *p* < 0.05. (**A**) Species Richness, (**B**) Shannon–Wiener, (**C**) Evenness.

A total of 3600 ASVs (Amplicon Sequence Variants) were obtained from all the DNA soil samples (n = 12). Rarefaction curves showed that the sequencing depth was sufficient to cover the fungal diversity from all agrosystems, as they all reached roughly saturation (Figure S1). Richness, Shannon–Wiener diversity index, and Evenness were calculated using the normalized data after rarefaction. Richness was similar in both management systems, but the Shannon–Wiener diversity index and Evenness were significantly higher in OF (Figure 1).

### 3.3. Soil Fungal Composition in Study Sites and Management Systems

**Figure 2:**
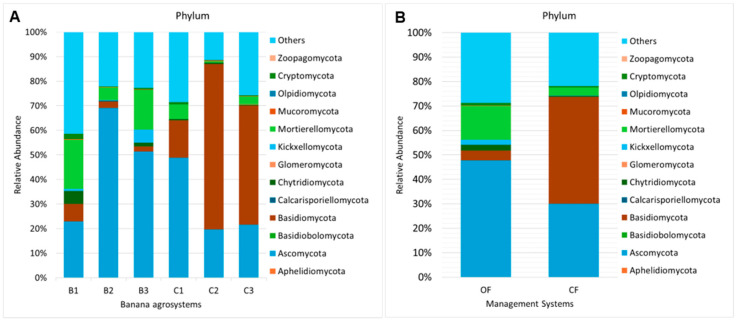
Mean relative abundance of phyla in (**A**) each banana agrosystem and (**B**) each management system.

Figure 2A shows the relative abundance of different phyla in the six agrosystems, while Figure 2B shows the relative abundance of phyla in the two management systems (OF and CF). The soil fungal communities from OF were dominated by the phyla Ascomycota (47.73%), followed by Mortierellomycota (13.84%), Basidiomycota (3.93%), and Chytridiomycota (2.39%). The three agrosystems from OF followed a similar pattern for the main phyla, differing only in abundance, and there were more Kickxellomycota (5.20%) than Basidiomycota (2.05%) and Chytridiomycota (1.63%) in B3. The soil fungal communities from CF were dominated by Basidiomycota (43.69%), followed by Ascomycota (30.03%) and Mortierellomycota (3.26%). However, the agrosystem C1 differs from C2 and C3, with Ascomycota dominating the community (48.84%), followed by Basidiomycota (15.25%) and Mortierellomycota (5.86%).

**Figure 3:**
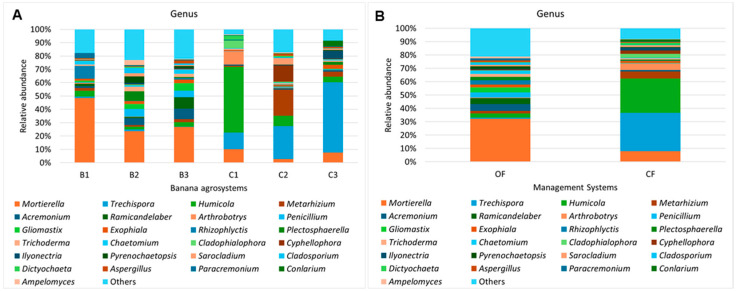
Mean relative abundance of genera in (**A**) each banana agrosystem and (**B**) each management system. Graphics discriminate the top 25 genera.

Figure 3 discriminates the top 25 genera that appear in each agrosystem (Figure 3A) and each management system (Figure 3B). In the three agrosystems under OF, *Mortierella* was the genus with higher relative abundance, representing 48.56% of total genera identified for B1, 23.54% in B2, and 26.77% in B3 and a mean of 32.12% in OF. The other genera found for OF agrosystems were represented in less than 10%. In CF agrosystems, there was a different distribution of the main genera; however, it is evident that *Trechispora* was well represented in the three agrosystems, with a mean of 28.81% in CF, followed by *Humicola* (25.79%) and *Mortierella* (7.86%). The agrosystem C1 was the one differing the most, with *Humicola* representing 49.35% of the total identified genera, followed by *Trechispora* (12.46%) and *Mortierella* (10.15%). The main genera for agrosystem C2 were *Trechispora* (24.71%), *Metarhizium* (19.52%), and *Cyphellophora* (12.22%). And finally, the main genera for agrosystem C3 were *Trechispora* (52.72%), *Mortierella* (7.58%), and *Ilyonectria* (5.93%). Due to the importance of this genus for banana plantations, the relative abundance of *Fusarium* was also analyzed; however, it represented mostly less than 1% of the total abundance in agrosystems from both management systems. There was an exception for C2 (1.37%) (Table S1).

**Figure 4:**
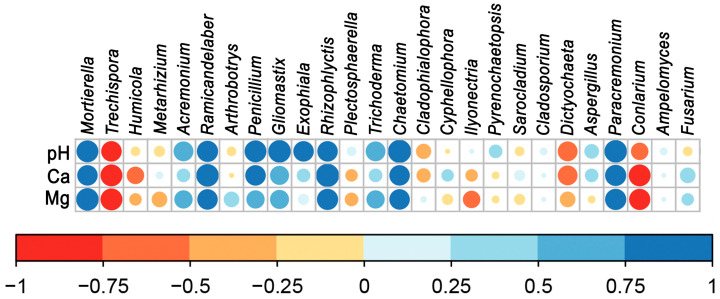
Observed Spearman’s correlations between the top 25 genera (+*Fusarium*) and pH, Mg, and Ca. Size of circles: the larger the circle, the smaller the *p*-value.

The indirect influence of management systems was evaluated through Spearman’s correlation between the top 25 genera with pH and Mg, which were significantly different between OF and CF, and we added Ca that was higher in OF, although not statistically different from CF and the genus *Fusarium* given its importance for banana agrosystems (Figure 4). Positively significant correlations were observed between pH and the genera *Mortierella* (*p* < 0.05), *Ramicandelaber* (*p* < 0.05), *Penicillium* (*p* < 0.05), *Gliomastix* (*p* < 0.01), *Chaetomium* (*p* < 0.01), and *Paracremonium* (*p* < 0.01) and no negatively significant correlations. The micronutrient Mg was positively correlated with *Mortierella* (*p* < 0.01), *Rhizophlyctis* (*p* < 0.05), and *Paracremonium* (*p* < 0.05) and negatively correlated with *Trechispora* (*p* < 0.05) and *Conlarium* (*p* < 0.05). For Ca, positively significant correlations with *Rhizophlyctis* (*p* < 0.01) and *Paracremonium* (*p* < 0.05) were found, and negatively significant correlations were found with *Trechispora* (*p* < 0.05) and *Conlarium* (*p* < 0.05). Finally, the genus *Fusarium* did not show any significant correlation with the three soil variables.

### 3.4. Differences in Functional Groups of OF and CF Management Systems

**Figure 5:**
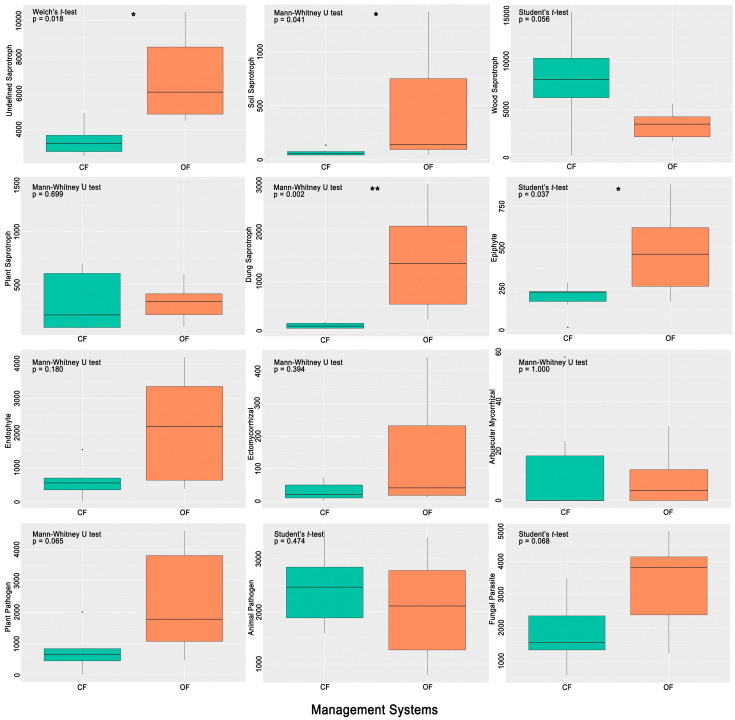
Variation of the abundance of fungal functional groups in each management system, conventional farming (CF) and organic farming (OF). The significant difference between CF and OF is labeled with * *p* > 0.05 or ** *p* > 0.01, Student’s *t*-test (*p* < 0.05), Welch’s *t*-test (*p* < 0.05) or Mann–Whitney U test (*p* < 0.05), depending on if homogeneity of variance and normality of the data were verified or not.

Using the FUNGuild database, it was possible to link 690 ASVs to a probable or highly probable ecological function. Figure 5 shows the variation in the abundance of each ecological function in both management systems. The agrosystems from OF showed a significantly higher abundance of fungi that were linked to unidentified (Welch’s *t*-test, *p* < 0.05), soil (Mann–Whitney U test, *p* < 0.05), and dung (Mann–Whitney U test, *p* < 0.01) saprotrophs. On the other hand, the CF agrosystems appeared to have more wood saprotrophs; however, the difference from OF is not statistically significant. Regarding symbiotic fungi, the six agrosystems showed more epiphytes and endophytes than ectomycorrhizal and arbuscular mycorrhizal fungi. Epiphytes were significantly higher in OF than in CF (Student’s *t*-test, *p* < 0.05). The total abundance of arbuscular mycorrhizal fungi did not exceed 60 reads in any agrosystem. There was no significant difference between OF and CF for pathogenic fungi.

PERMANOVA was used to determine the effect of management practices and soil properties on soil fungal functional communities (Table 3). Management systems (MS) is the factor that most affects the fungal functional communities, explaining 38.71% of the variance (*p* < 0.001), followed by the macronutrient K (20.57%, *p* < 0.001), OM (8.17%, *p* < 0.1), *p* (7.34%, ns), and pH (4.08%, ns).

**Figure 6:**
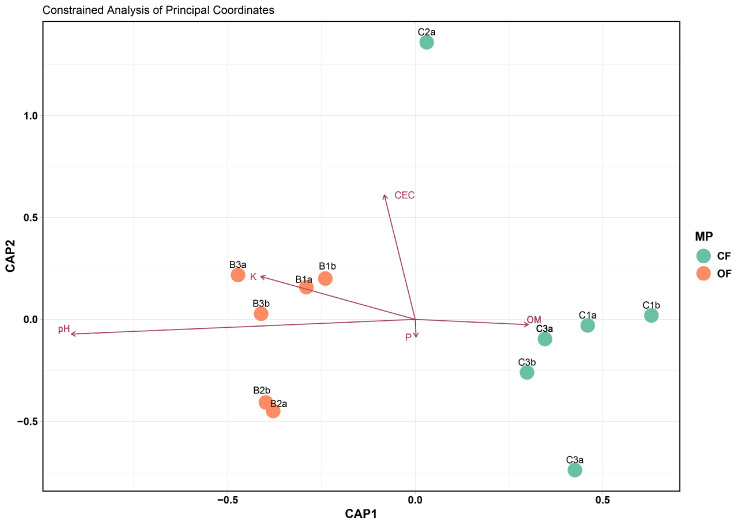
CAP based on the Bray–Curtis dissimilarity distance matrix of soil fungal functional communities in two management systems, conventional farming (CF) and organic farming (OF), and the relation with soil physicochemical properties.

The distribution of the soil fungal functional communities from the six agrosystems (in duplicate) is shown in CAP (Figure 6). Samples from OF and CF agrosystems are well separated, with K positively correlated with most of the OF samples and OM positively correlated with most of the CF samples, corroborating the PERMANOVA results.

## 4. Discussion

The rising global population has sharply increased food demand, prompting farming practices like intensive mechanization and heavy chemical use [10,47,48]. These methods, however, have led to environmental degradation and soil health concerns [10,48,49]. To achieve sustainable growth of food production, it is essential to decrease the agriculture’s environmental footprint [47]. Organic farming is a holistic system that favors good farm management practices that preserve and improve the ecosystem’s health by promoting biodiversity, biological cycles, and biological activity in the soil [23,50].

Soil fungi play a central role in agrosystems. These microorganisms can participate in organic matter decomposition, nutrient and water delivery, and plant protection or interact with animals and plants as pathogens or as limiting factors [51]. However, fungal communities may be influenced by several factors, including land use [52,53,54]. In this study, we reported the differences between soil fungal communities of banana agrosystems under OF and CF. In addition, the indirect impact of the management practices adopted was analyzed through the soil physicochemical properties found in the management systems used in the agrosystems.

A significant shift in the structure of fungal communities and functional groups was observed between management systems. OF showed significantly higher Shannon–Wiener diversity and Evenness and a higher abundance of epiphytes and unclassified soil and dung saprotrophs. Inconsistent results about the impact of OF and CF in fungi alfa diversity have been found in the literature [53,55,56]. This could be due to the different management practices applied for different crops. Li et al. [57] found that two organic fertilizers significantly increased the pH and other soil properties in comparison to chemical fertilizers applied to banana plantations. In addition, fungal quantification was higher with organic fertilizers, and fungal compositions changed. An experiment conducted in soils in a tropical region also found an increase in pH in organic systems and high amounts of Mg in nature management, i.e., organic management based on green manure, following natural farming principles proposed by the philosopher Mokiti Okada [58]. In our study, OF improved the pH and Mg when compared with the CF management system but had no significant differences for the other soil physicochemical properties. Regarding fungal composition, OF and CF followed different patterns, and soil physicochemical properties had an important contribution. The pH and Mg were positively associated with the increase in genera from *Mortierellomycota*, *Kickxellomycota*, *Chytridiomycota*, and *Ascomycota*. Li et al. [57] also found a higher abundance of *Mortierellomycota*, *Kickxellomycota*, and *Chytridiomycota* with the organic fertilizer that increased the pH the most and improved other soil properties, in addition it was the treatment with the best results for productivity. Another study, where different rates of Mg were applied to the soil, reported an increase in *Kickxellomycota* with Mg increment; however, *Mortierellomycota* decreased, which differed from our results [59]. The phylum *Ascomycota* was found to be dominant in healthy banana rhizospheres soils in a previous study [60], which is in line with our results, but this phylum was also linked to an increase in *Fusarium* wilt in other studies [29,61]. Although *Fusarium* wilt is a major concern for banana production [7,8,9], in our study, the genus *Fusarium* was in low abundance in both management systems (OF and CF), representing less than 1% of total abundance. Major genera belonging to *Ascomycota* in the OF management system were *Acremonium*, *Penicillium*, *Gliomastix*, *Humicola*, and *Chaetomium*. These genera are all classified as saprotrophs in the FUNGuild database [46], which explains the higher abundance of undefined soil and dung saprotrophs compared to the CF management system. Some species of these genera are also symbionts (epiphytes and endophytes) or pathogens [46]. However, there were no statistically significant differences from CF for harmful fungi, such as plant or animal pathogens. The genus *Mortierella* dominated the agrosystems. Several studies also found this genus to be dominant or in high abundance in healthy banana systems, in systems where organic practices are applied, and also in the core fungal of banana´s microbiome [30,57]. *Mortierella* species are considered beneficial fungi and indicators of healthy soils because of their great capacity to decompose plant organic matter and aromatic hydrocarbons or their ability to assist plants and mycorrhizal fungi in nutrient acquisition [62,63]. On the other hand, mycorrhizal fungi, one of the most important functional groups in soils [64], were in low abundance in both management systems, especially the arbuscular mycorrhizal fungi (AMF). Mycorrhizal fungi improve soil structure through aggregation and interaction with plant roots, exchanging nutrients and improving their growth and resistance to biotic and abiotic stresses [65]. Although banana cropping systems show low soil disturbance compared to other annual crops, the low abundance of AMF is probably due to the high amounts of phosphorus found in banana agrosystems analyzed in this study. A study conducted in Rwanda analyzed the AMF communities in 188 banana fields and found that the frequency and intensity of colonization were negatively correlated with P content [66]. Similar results were found by Jefwa et al. [67] in banana plantations in Kenya. Other fungal symbionts were found in banana agrosystems in this study. Epiphytes and endophytes were in higher abundance than mycorrhizal fungi, and OF showed a significantly higher abundance of epiphytes than CF. Interactions between these fungi and plants are complex and include commensalism, parasitism (opportunistic), and mutualism, depending on environmental factors and the genetic, nutritional, and development stages of the plant [68,69]. However, they may offer several benefits to plants and the ecosystem, such as increasing plant growth and resistance to environmental stresses or remediating contaminated sites [70]. In this study, the most abundant ASVs classified as epiphytes and endophytes were from the genera *Acremonium*, *Plectosphaerella*, *Trichoderma*, and *Cladosporium*. The low abundance of the genus *Fusarium* may be related to the presence of these functional groups, as several studies report the antagonism or induction of resistance in plants against pathogens invasion [71,72].

To conclude, the OF management system adopted in banana plantations from Madeira Island showed a positive impact on the structure and function of fungal communities, enhancing the alfa diversity and presence of several beneficial fungi and functional groups. Nevertheless, care must be taken with the input of macronutrients, especially phosphorus, to allow the colonization of soil and plants by important fungi such as AMF.

## Figures and Tables

**Table 1:** General information on the 6 banana plantations under study.

Agrosystem ID	Management System	Coordinates	Age of the Agrosystem	Altitude (a.s.l.)
B1	OF	32.6678705 −16.9617026	>50 years; OF certification since 2012	152
B2	OF	32.7114335 −17.1464224	>50 years; OF certification since 2015	217
B3	OF	32.6466043 −16.8831825	>20 years; OF certification since 2017	90
C1	CF	32.763168 −17.2333474	>50 years	13
C2	CF	32.6878779 −17.1020215	>20 years	214
C3	CF	32.6983176 −16.7830542	>50 years	179

**Table 2:** The effect of different management systems on soil physicochemical properties.

Soil Properties	Management System		*t*-Test	Mann–Whitney U
	OF	CF		
pH	6.57 ± 0.55	4.27 ± 0.21	**	-
OM (%)	3.83 ± 1.32	4.56 ± 1.22	ns	-
P (ppm)	1206 ± 503	1007 ± 478	ns	-
K (ppm)	1480 ± 250	1200 ± 208	ns	-
Ca (meq/100 g)	27.37 ± 13.45	8.63 ± 1.36	ns	-
Mg (meq/100 g)	12.77 ± 3.59	6.00 ± 1.30	*	-
Na (meq/100 g)	0.70 ± 0.36	0.43 ± 0.12	ns	-
CEC (meq/100 g)	56.57 ± 12.07	60.10 ± 28.96	ns	-
SD (%)	79.67 ± 35.22	34.33 ± 11.55	-	ns
NO_3_-N (ppm)	35.00 ± 34.64	68.33 ± 54.85	ns	-
NH_4_-N (ppm)	4.13 ± 2.66	5.00 ± 1.13	ns	-
Cu (ppm)	5.17 ± 2.75	6.67 ± 4.65	ns	-
Zn (ppm)	9.33 ± 2.93	7.17 ± 2.93	ns	-
Mn (ppm)	16.50 ± 1.50	16.50 ± 0.50	ns	-
Fe (ppm)	50.00 ± 5.00	53.33 ± 2.89	ns	-

OM—organic matter; CEC—cation exchange capacity; SD—saturation degree; macro- and micronutrients (P, K, Ca, Mg, Na, NO_3_-N, NH_4_-N, Cu, Zn, Mn, Fe); OF—organic farming; CF—conventional farming. Values (mean ± standard deviation n = 3); ns—non-significant differences between management practices; significant levels: ** *p* < 0.01; * *p* < 0.05.

**Table 3:** PERMANOVA table generated based on Bray–Curtis dissimilarity distance matrix, showing the significant effect of management systems and soil physicochemical properties in soil fungal functional communities. The number of permutations = 9999. Terms are added sequentially (first to last).

	Df	SumOfSqs	R2	F	Pr(>F)	
MS	1	0.108915	0.38709	10.9904	0.0001	***
pH	1	0.011481	0.04080	1.1585	0.3623	ns
OM	1	0.022982	0.08168	2.3190	0.0760	.
P	1	0.020652	0.07340	2.0839	0.1063	ns
K	1	0.057882	0.20571	5.8408	0.0009	***
Residual	6	0.059460	0.21132			
Total	11	0.281370	1.00000			

MS—management systems; OM—organic matter; macronutrients (P, K); ns—the effect is non-significant; significant levels: *** *p* < 0.001; . *p* < 0.1.

## Data Availability

Research data used in this article are part of a wider data collection deposited in BioProject database: https://www.ncbi.nlm.nih.gov/sra/PRJNA1107202, accessed on 10 November 2024, accession reference: PRJNA1107202.

## Supplementary Materials

The following supporting information can be downloaded at: https://www.mdpi.com/article/10.3390/microorganisms12112372/s1, Figure S1: Rarefaction curves; Table S1: Abundance of *Fusarium*.
