# Supplementary material for: Organic Farming Enhances Diversity and Recruits Beneficial Soil Fungal Groups in Traditional Banana Plantations
Source: Microorganisms. 2024 Nov 20;12(11):2372. doi: 10.3390/microorganisms12112372 (PMC11596534; doi:10.3390/microorganisms12112372)
Supplement: Supplementary file 1 [file microorganisms-12-02372-s001.zip › microorganisms-3272293-supplementary.pdf]

Supplementary Material

Materials and Methods

Site description and soil sampling

Detailed soil physicochemical properties procedures (as referenced in the article, Ragonezi et al. [28], and Temminghoff and Houba [29]).

The pH and electrical conductivity (EC) were measured using a probe on the final aqueous aqueous extracts of the soil. Organic matter content (OM) was determined by digesting the soil sample with sodium dichromate in sulfuric acid followed by titration with ferrous ammonium sulphate solution (Walkley and Black method). Cation exchange capacity (CEC) and Saturation degree (SD) were determined through percolation of the soil samples with an ammonium acetate solution at pH 7.0.

The concentration of the macronutrients NO3-N and NH4-N were determined using a continuous flow analyser after distilled water extraction. P and K concentrations were measured by extraction in an ammonium lactate solution. The micronutrients content, Ca, Mg, Na, Cu, Zn, Mn and Fe was determined through atomic absorption spectrometry. Briefly, the samples were digested with hydrochloric acid for the quantification of Cu, Fe, Mg, Mn and Zn, while Ca, Mg and Na were extracted using bidistilled water.

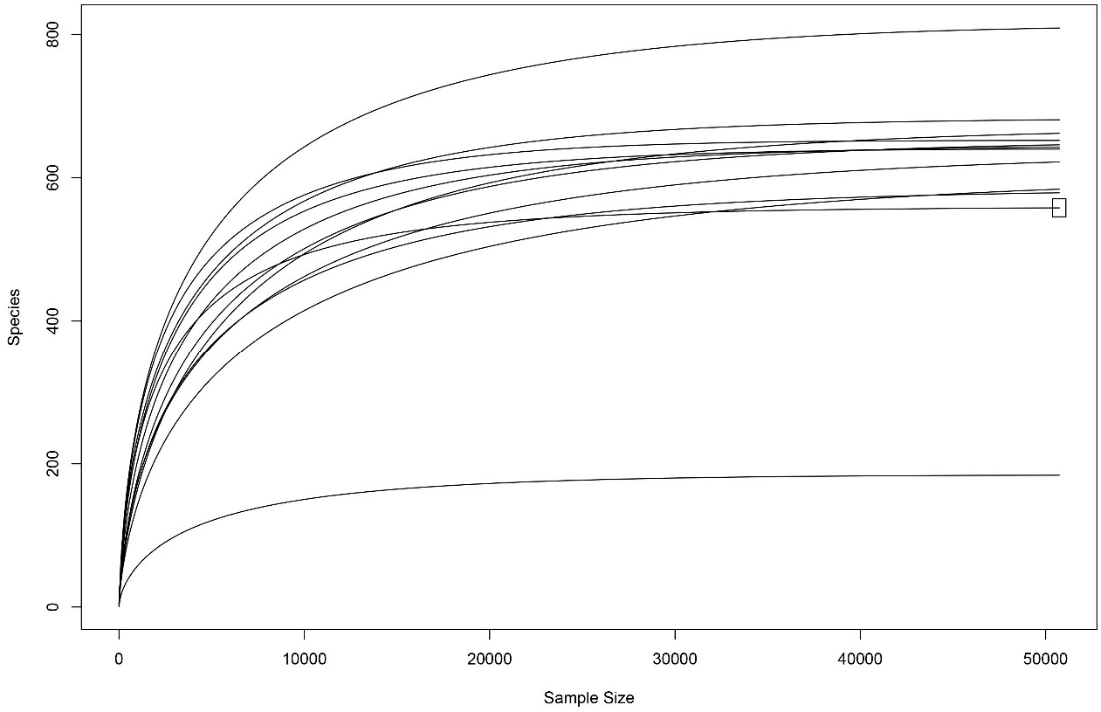

Figure S1. Rarefaction curves of the 6 agrosystems (in duplicates).

Table S1. Average abundance and relative abundance of the genus *Fusarium* in each management system and agrosystem.

***Fusarium* spp.**			
		**Mean Abund. (ASV's)**	**Relative Mean Abund.**
**Management Systems**	OF	92	0.38
	CF	68	0.32
**Agrosystems**	B1	82	0.39
	B2	135	0.64
	B3	60	0.19
	C1	24	0.08
	C2	162	1.37
	C3	18	0.08
